# Intraocular Lens Calculation Concept Based on Aphakic Refraction—Considerations on a Cornea Model With Two Refracting Surfaces

**DOI:** 10.1007/s44402-026-00109-0

**Published:** 2026-06-16

**Authors:** Achim Langenbucher, Nóra Szentmáry, Alan Cayless, Peter Hoffmann, Jascha Armin Wendelstein

**Affiliations:** 1 Department of Experimental Ophthalmology, Saarland University, Homburg, Germany; 2 Department of Ophthalmology, Semmelweis University, Budapest, Hungary; 3 School of Physical Sciences, The Open University, Milton Keynes, UK; 4 Augen- und Laserklinik Castrop-Rauxel, Castrop-Rauxel, Germany; 5 Department of Ophthalmology, LMU Klinikum, Munich, Germany

**Keywords:** Aphakic refraction, ELP prediction model, IOL power calculation, Two-surface cornea model, Vergence calculation

## Abstract

**Purpose:**

To develop a concept for predicting the power of an intraocular lens (IOL) and the spectacle refraction after cataract surgery based on aphakic refraction (REFa) using vergence transforms with a two-surface cornea model.

**Methods:**

This simulation study is based on a large dataset originally used in IOLCon (www.IOLCon.org) for optimising lens constants. Proxy values for REFa calculated from the available information in this dataset were used to develop a strategy for predicting the effective lens position (ELP). A variety of models, including linear, quadratic, sigmoidal and two stepwise linear models, were evaluated. Using this ELP, vergence transform techniques were applied to derive the IOL power for a given target refraction or the spectacle refraction for a given IOL power. This concept was applied to subsets of the IOLCon dataset with the Hoya Vivinex and the Bausch & Lomb enVista lenses to show the efficiency of the calculation.

**Results:**

After evaluating the ELP prediction models on the 22,577 IOLCon data, it was decided to use the sigmoidal model for calculating the ELP corrected by a linear term for the *A* constant. Compared with the fully disclosed Castrop formula, the predicted ELP showed a root-mean-squared deviation of 0.221/0.221 mm with the Vivinex/enVista lens and the predicted IOL power showed a root-mean-squared deviation of 0.369/0.375 D.

**Conclusions:**

Where biometric data for a classical IOL power calculation are unavailable, this vergence-based calculation concept may help to identify the appropriate IOL power and to predict the spectacle refraction after cataract surgery. A clinical study with ‘real’ REFa measurements is required to validate these results.

Key Points

- *Aphakic refraction enables intraocular lens power calculation without standard biometry*: using vergence principles and a two-surface cornea model, the effective lens position can be predicted directly from aphakic refraction, independent of axial length, anterior chamber depth or keratometric assumptions.
- *Sigmoidal effective lens position model achieves high accuracy across a large dataset*: a sigmoidal effective lens position prediction function, derived from 22,577 clinical cases and corrected by a linear *A* constant term, yields an effective lens position deviation of 0.22 mm and an intraocular lens power deviation of approximately 0.37 D.
- *Vergence-based framework allows bidirectional prediction*: the method supports both (a) computing the intraocular lens power required for any target refraction and (b) predicting the postoperative spectacle refraction for a given intraocular lens power—using a single, consistent optical model.

## Background

In phakic eyes, intraocular lens (IOL) power calculation is normally based on biometric data such as axial length (AL) and keratometry. Additional parameters such as central corneal thickness (CCT), phakic anterior chamber depth (ACD), lens thickness (LT) or the horizontal corneal diameter (CD) could be used to enhance the predictability of the IOL power (IOLP) or the axial position in the pseudophakic eye, i.e., the effective lens position (ELP) [1–4]. In this context, keratometry refers to the measured radius of curvature of the corneal front surface (Ra), converted to corneal power (PK) using a keratometer index (nK). The IOLP is the refractive power of a thin replacement lens, characterising the refractive properties of the thick IOL implant. This is similar to the IOLP as labelled by the manufacturer, although not directly equivalent since it depends on the location of the image-side principal plane, which is unknown for most IOL models [1, 3, 5, 6].

However, it is known that biometry may be unreliable in some situations and sometimes cannot even be measured, e.g., in situations with dense cataract where optically dense media impede optical biometry measurement. Therefore, concepts are required to predict the IOLP for a specific target refraction (TR) or the spectacle refraction (REF) for a given IOLP [7, 8]. Additionally, keratometric measures of Ra may be unreliable in eyes with a history of corneal surgery, and in such cases would not represent corneal power accurately [1, 2].

Several techniques have been described for measuring aphakic refraction (i.e., refraction in the aphakic eye), either preoperatively or intraoperatively after extraction of the opaque crystalline lens. Such methods include manual streak retinoscopy or automated measurements such as video refractometry, autorefractometry or wavefront aberrometry [7–12]. Given the large ametropia of the aphakic eye, special care should be given to the location of the correcting spectacles, with even small variations in the vertex distance (VD) leading to significant changes in the measured aphakic refraction [1, 5].

Using the aphakic refraction and corneal power data, it is possible to derive either the IOLP for a given TR or vice versa, and the expected spectacle refraction for a given IOLP. The key point is the prediction of the ELP, since standard techniques using biometric data such as AL, ACD, LT or CD cannot be applied [8–13]. Therefore, strategies are required to circumnavigate biometric data and predict the ELP from the aphakic refraction. Another important point is that the calculation concept should be independent of the keratometer refractive index, as this might provide an incorrect conversion from Ra to PK in situations where the ratio of corneal front to back surface radius or corneal thickness does not match the underlying cornea model, especially in eyes with a history of corneal refractive surgery, penetrating/lamellar keratoplasty, ectatic corneal diseases or corneal oedema [1, 2, 5].

The purpose of the present study was to develop a concept to predict

- the IOLP for a given TR,
- the spectacle refraction for a given IOLP and
- the AL of the eye

based on aphakic refraction and a cornea model with corneal front and back surface curvature and corneal thickness.

## Methods

The calculation strategy in this simulation study consists of the following five steps: (A) a strategy to derive the aphakic refraction from the available biometric data. This step is only required in this simulation study as a proxy for actual aphakic refraction measurements, which were not available in this database; (B) Prediction of AL based on aphakic refraction. This calculation step could be of assistance with e.g., 3rd generation IOLP formulae such as the Sanders-Retzlaff-Kraff Theoretical (SRK/T) [13], Hoffer *Q* [14] or Holladay 1 [15] in cases where measurements of the AL are unavailable; (C) Prediction of the axial lens position from the aphakic refraction and the *A* constant, which is a key element for the entire lens power calculation; (D) Prediction of the lens power for a TR based on the ELP and (E) Prediction of the spectacle refraction for a given lens power based on the ELP.

For all calculations and especially for the development of the ELP prediction model, a large clinical dataset was used, containing 36,362 biometric measurements, which have been uploaded in an anonymised fashion by several surgical centres between November 2017 and November 2025 to our IOLCon database (www.IOLCon.org) for lens constant optimisation. Only biometric measures from the IOLMaster 700 (Carl-Zeiss Meditec, zeiss.com) were considered. Data tables were reduced to the relevant parameters required for the data analysis, consisting of the following measurements: Ra, corneal back surface radius (Rp), both in mm, the AL in mm, CCT in mm, ACD in mm measured from the corneal epithelium to the lens and the central thickness of the crystalline lens (LT) in mm. Incomplete records (e.g., missing Rp, CCT or LT) were excluded from the analysis.

The local Institutional Review Board provided a waiver for this retrospective study (Ethikkommission der Ärztekammer des Saarlandes, registration number 157/21) and informed consent from the patients was not required. The data were transferred to MATLAB (MATLAB 2023a, mathworks.com) for further processing.

### Step-by-Step Approach of The Calculation Strategy

(A) Using classical vergence transform, the aphakic refraction (REFaphakic) at a reference plane for a distance VD in front of the corneal apex and using a two-surface cornea model is given by

1 $${REFaphakic}=\frac{1}{\displaystyle\frac{1}{\displaystyle\frac{1}{\displaystyle\frac{1}{\frac{n}{{AL}-{CCT}}-\displaystyle\frac{n-{nC}}{{Rp}}}+\displaystyle\frac{{CCT}}{{nC}}}-\frac{{nC}-1}{{Ra}}}+{VD}}$$

where *n* = 1.336 refers to the refractive index of the aqueous or vitreous humour and nC = 1.376 as the refractive index of the cornea. As measurements for aphakic refraction were unavailable, these back-calculated values were used as a proxy.

(B) Using REFaphakic, the AL can be predicted from the vergence at the corneal back vertex plane by

2 $${ALpred}={CCT}+\frac{n}{\displaystyle\frac{1}{\displaystyle\frac{1}{\frac{{REFaphakic}}{1-{REFaphakic}\cdot {VD}}+\displaystyle\frac{{nC}-1}{{Ra}}}-\displaystyle\frac{{CCT}}{{nC}}}+\frac{n-{nC}}{{Rp}}}$$

(C) The axial position (ELP) of the IOL is normally predicted from biometric parameters such as AL, ACD or LT, which were assumed to be unavailable for the calculation scheme. To overcome this lack of data, the large IOLCon dataset of clinical data (preoperative biometry, model and power of the implanted lens together with the respective formula constants for the Castrop formula (C, H and R) and the SRK/T *A* constant (*A*) were used as listed in www.IOLCon.org). The Castrop formula [1, 2, 4] was selected as it is based on a two-surface cornea model (identical to the cornea model used in the present study), the ELP mostly matches the real axial IOL position due to a realistic cornea model and its architecture is fully disclosed. Various prediction models were tested to characterise these ELP data in terms of REFaphakic and a function $f\left(A\right)$ of the *A* constant of the respective lenses, using values also listed on www.IOLCon.org. These prediction models included:

a linear model

3a $${ELP}=f\left(A\right)+p0+p1\cdot {REFaphakic},$$

a quadratic model

3b $${ELP}=f\left(A\right)+p0+p1\cdot {REFaphakic}+p2\cdot {{REFaphakic}}^{2},$$

a sigmoidal model

3c $${ELP}=f\left(A\right)+a+(b-a)/(1+\exp (-4\cdot \log (3)\cdot ({REFaphakic}-c)/d))$$

a piecewise model with one changepoint (*k*)

3d $${ELP}=f\left(A\right)+\left\{\begin{array}{rcl}p01+p11\cdot {REFaphaki}c & {for} & {REFaphakic} \; < \; k\\ p02+p12\cdot {REFaphakic} & {for} & {REFaphakic}\ge k\end{array}\right\},$$

and a piecewise model with two changepoints (*k* and *l*)

3e $${ELP}=f\left(A\right)+\left\{\begin{array}{rcl}p01+p11\cdot {REFaphakic} & {for} & {REFaphakic} \; < \; k\\ p02+p12\cdot {REFaphakic} & {for} & k\le {REFaphakic} \; < \; l\\ p03+p13\cdot {REFaphakic} & {for} & {REFaphakic}\ge l\end{array}\right\}.$$

The parameters $p$01 – $p$13, $a$ to $d$ and the changepoints $k$ and $l$ are listed for each model in Table 1, and $f\left(A\right)$ is defined according to the assumption that the *A* constant produces a linear shift of the lens position as:

4 $$f\left(A\right)=m0+m1\cdot A.$$

**Table 1:** Fit models to predict the axial lens position of the intraocular lens (effective lens position, ELP) from the aphakic refraction (REFaphakic).

Model	Fit parameter	95% confidence interval	RMS fit error in mm	Coefficient of determination *R*²
Linear model	*p*0: 8.2380 *p*1: −0.0591	[8.220 to 8.257] [−0.0594 to −0.0588]	0.2169	0.390
Quadratic model	*p*0: 2.9060 *p*1: 0.1333 *p*2: −0.0017	[2.725 to 3.088] [0.1268 to 0.1399] [−0.0018 to −0.0017]	0.2140	0.403
Sigmoidal model	*a*: 5.601 *b*: 3.638 *c*: 60.74 *d*: 30.28	[5.588 to 5.673] [3.580 to 3.696] [60.35 to 61.14] [29.70 to 30.85]	0.2127	0.422
Linear piecewise 1 changepoint	*p*01: 5.4723 *p*11: −0.0390 *p*02: 5.0704 *p*12: −0.1173 *k*: 10.31	[5.463 to 5.481] [−0.0400 to −0.0379] [5.012 to 5.103] [−0.1182 to −0.1164] [10.27 to 10.36]	0.2144	0.410
Linear piecewise 2 changepoints	*p*01: 5.4854 *p*11: −0.0409 *p*02: 5.0462 *p*12: −0.1376 *p*03: 5.3074 *p*13: −0.0510 *k*: 10.75 *l*: 14.05	[5.477 to 5.494] [−0.0418 to −0.0399] [5.0388 to 5.0501 [−0.1391 to −0.1361] [5.3011 to 5.3110] [−0.0549 to −0.0470] [10.71 to 10.78] 13.98 to 14.12]	0.2141	0.418

The table lists the fit parameters together with their 95% confidence intervals, the root-mean-squared fit error and the coefficient of determination *R*². Note that this ELP prediction is independent of the lens geometry or characteristics. A separate correction term *f*(*A*) is used to consider the *A* constant of the lens.

*RMS* root mean square.

All of the prediction models mentioned above were optimised using a nonlinear iterative optimisation strategy (Trust-Region-Reflective algorithm) based on minimising the root-mean-squared fit error between the ELP calculated with the Castrop formula and the ELP predicted from REFaphakic and the *A* constant [16–18].

(D) The predicted IOLP is derived from a classical vergence transform from REFaphakic, TR and the predicted ELP by

5 $$\begin{array}{cc}{IOLP}= & \displaystyle\frac{1}{\displaystyle\frac{1}{\displaystyle\frac{1}{\displaystyle\frac{1}{\displaystyle\frac{{REFaphakic}}{1-{REFaphakic}\cdot {VD}}+\frac{{nC}-1}{{Ra}}}-\frac{{CCT}}{{nC}}}+\frac{n-{nC}}{{Rp}}}-\frac{{ELP}-{CCT}}{n}}-\\ & -\displaystyle\frac{1}{\displaystyle\frac{1}{\displaystyle\frac{1}{\displaystyle\frac{1}{\displaystyle\frac{{TR}}{1-{TR}}+\frac{{nC}-1}{{Ra}}}-\frac{{CCT}}{{nC}}}+\frac{n-{nC}}{{Rp}}}-\frac{{ELP}-{CCT}}{n}}\end{array}.$$

(E) If it is assumed that a lens with a refractive power IOLP is placed at ELP behind the front corneal vertex, the resulting spectacle refraction REF at the spectacle plane can be predicted using a vergence transform by6

### Statistics

Descriptive statistics include the arithmetic mean, standard deviation, median and lower and upper boundaries of the 95% confidence intervals. Correlations between the ELP derived from the Castrop formula [1, 2, 4] and the predicted ELP from aphakic refraction and between IOLP derived from the Castrop formula [1, 2, 4] and the predicted IOLP from aphakic refraction were performed using the Spearman rank correlation coefficient *R* (coefficient of determination *R*²). For the ELP prediction models, the model parameters were provided together with the estimation of their 95% confidence intervals. The root-mean-squared (RMS) prediction error was used as a quality metric for the model performance. *p* Values < 0.05 were considered statistically significant.

## Results

**Fig. 1:**
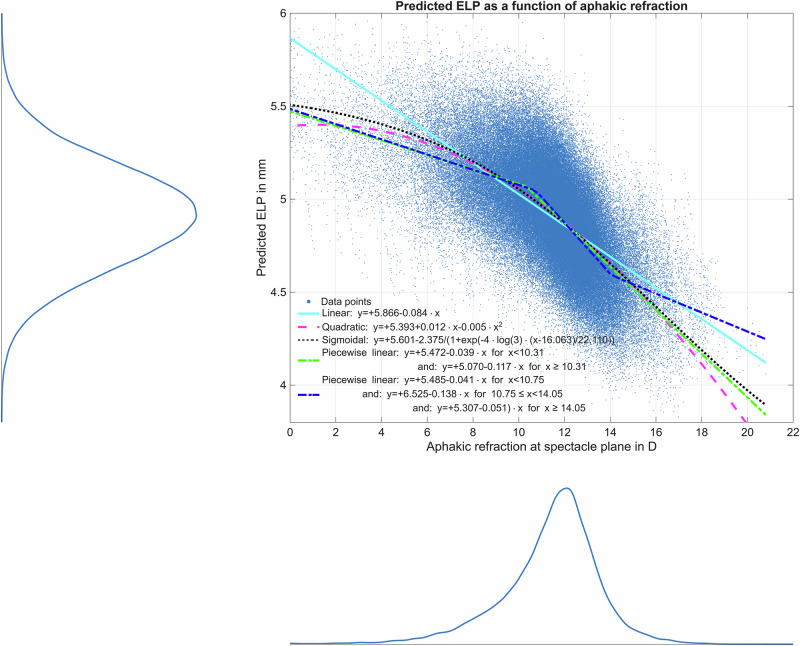
Scatter-histogram plot showing the relationship between the back-calculated aphakic refraction REFaphakic and the axial position of the intraocular lens (effective lens position, ELP) predicted with the Castrop formula in *N* = 22,577. Data derived from IOLCon (IOLCon.org). The findings show large scatter in ELP, mostly for myopic eyes (with lower REFaphakic values and higher ELP values) and some saturation effects in ELP for both lower and higher ELP values. Fits for ELP as a function of REFaphakic are shown in the following colours: linear (cyan), quadratic (magenta), sigmoidal (black) and piecewise linear models with one (green) and two changepoints (blue). The fit parameters are shown in the main plot. D dioptres.

Of the *N* = 36,362 data records uploaded to IOLCon for lens constant optimisation, *N* = 22,577 complete records were considered in this study. For these data, the ELP was calculated according to the Castrop formula using the appropriate formula constants *C*, *H* and *R* for the implanted lenses as listed in www.IOLCon.org. The proxy aphakic refraction was then derived using the formula shown in step (A), based on the biometric data AL, Ra, Rp, CCT and VD = 12 mm. This REFaphakic was used as a reference to develop a strategy for estimating the ELP value from the Castrop formula [1, 2, 4]. Figure 1 uses a scatter-histogram plot to show the relationship between REFaphakic (calculated according to step A) and ELP predicted with the Castrop formula. The data imply a large scatter in ELP, mostly for myopic eyes (with lower REFaphakic values and higher ELP values) and some saturation effects in ELP for both lower and higher ELP values. The corresponding fit parameters together with their 95% confidence intervals are shown, and the performance in terms of *R*² and the RMS) fit error for the fit models are listed in Table 1.

The sigmoidal fit showed the best performance with the lowest RMS fit error and some consideration of a saturation effect to avoid excessively low or high ELP values (in short or long eyes). The saturation effect refers to a situation where increases in an input variable initially lead to strong gains in output, but after a certain point, additional increases produce diminishing returns and eventually almost no change. A sigmoidal (S-shaped) prediction model captures this behaviour naturally. Therefore, it was decided to use this sigmoidal model for the estimation of the ELP from REFaphakic. To account for various lens designs and characteristics, a linear regression model was derived to correct the ELP prediction with an additive term including the *A* constant of the lens. In this context, *f*(*A*) = *m*0 + *m*1∙*A* reads

$$\begin{array}{ccc}m0=-75.76 & & [-76.47\,{to}-75.05]\\ & 95 {{{\rm{ \% }}}}\; {CI}\!\!: & \\ m1=0.6368 & & [0.6308\,{to}\,0.6428]\end{array}$$

with a RMS fit error of 0.283 mm and a coefficient of determination of *R*² = 0.173.

The performance of this calculation scheme was tested using two common, commercially available lenses, with clinical data available on www.IOLCon.org as examples: the XC1/XY1 Vivinex (Hoya Surgical Optics, hoyasurgicaloptics.com; Castrop constants *C*/*H*/*R* = 0.3230/0.1605/0.1193, *A* constant 119.192, *N* = 2883 datapoints) and enVista (Bausch and Lomb Surgical, bauschsurgical.com; *C*/*H*/*R* = 0.3688/0.1554/−0.1246, *A* constant = 119.275, *N* = 873 datapoints). For both lenses, the IOLP and the ELP were calculated according to the Castrop formula. REFaphakic was determined using the vergence transform as described in calculation step (A), and the ELP and the IOLP were predicted from REFaphakic and the *A* constant, based on the calculation scheme.

**Fig. 2:**
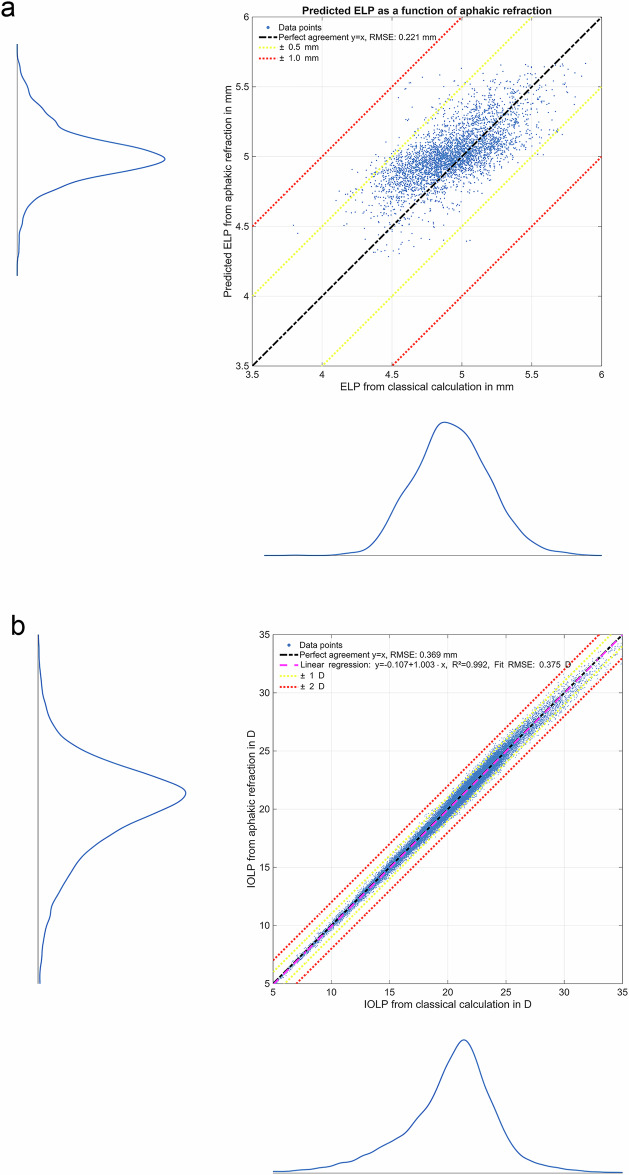
Simulated performance of the lens power calculation based on aphakic refraction (back-calculated from biometric measures using vergence transform) for the *Vivinex lens (Hoya Surgical Optics, hoyasurgicaloptics.com)*. **a** Agreement between the effective lens position (ELP) calculated with the Castrop formula and the ELP predicted from the present calculation scheme based on aphakic refraction. The black dash-dotted line indicates a hypothetical perfect match between the two methods, with yellow and red dashed lines for tolerance regions of ±0.5 and ±1.0 mm, respectively. The root-mean-squared deviation from the perfect match is shown in the plot (RMSE = 0.221 mm). **b** Agreement between intraocular lens power (IOLP) calculated with the Castrop formula and the IOLP predicted from the present calculation scheme based on aphakic refraction. The black dash-dotted line indicates a perfect match, with yellow and red dashed lines for tolerance regions of ±0.5 and ±1.0 D, respectively. The magenta dashed line indicates the linear regression line, which is very close to the perfect match. The root-mean-squared deviations from the perfect match (RMSE = 0.369 D) and from the linear regression line are listed in the plot (RMSE = 0.375 D), together with the parametric definition of the linear regression line (*y* ~ *a* ۰ *x* + *b*) and the coefficient of determination (*R*² = 0.992). D dioptres, RMSE root mean square error.

Figure 2 shows the simulated performance of the lens power calculation based on aphakic refraction for the Vivinex lens. Figure 2a displays the agreement between ELP calculated with the Castrop formula and the ELP predicted from the present calculation scheme based on aphakic refraction. Figure 2b displays the agreement between the IOLP calculated with the Castrop formula and the IOLP predicted from the present calculation scheme based on aphakic refraction.

**Fig. 3:**
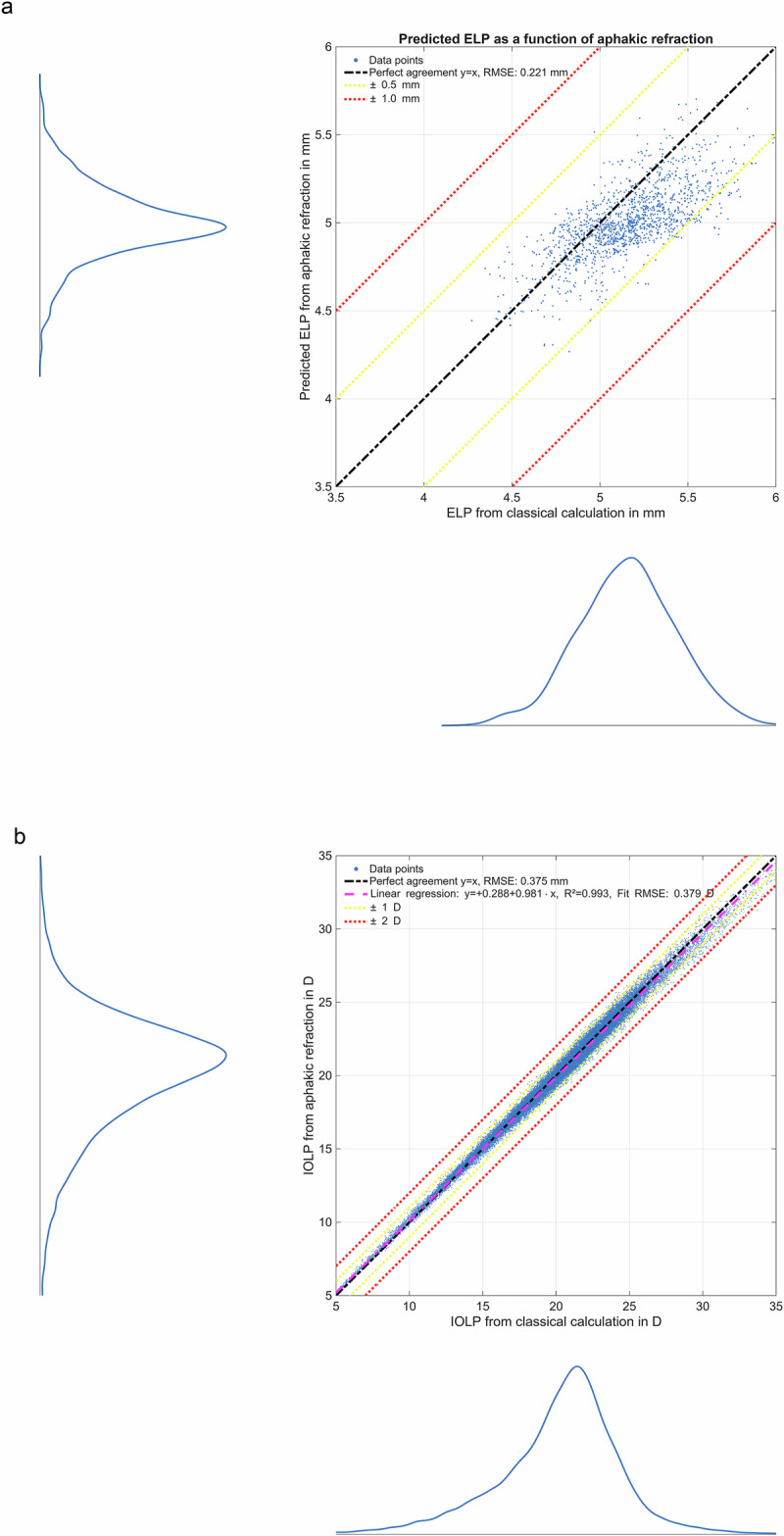
Simulated performance of the lens power calculation based on aphakic refraction (back-calculated from biometric measures using vergence transform) for the *enVista lens (Bausch and Lomb, bauschsurgical.com)*. **a** Agreement between the effective lens power (ELP) calculated with the Castrop formula and the ELP predicted from the present calculation scheme based on aphakic refraction. The black dash-dotted line indicates a hypothetical perfect match between the two methods, with yellow and red dashed lines for tolerance regions of ±0.5 and ±1.0 mm, respectively. The root-mean-squared deviation from the perfect match is shown in the plot (RMSE = 0.221 mm). **b** Agreement between IOLP calculated with the Castrop formula and the IOLP predicted from the present calculation scheme based on aphakic refraction. The black dash-dotted line indicates a perfect match, with yellow and red dashed lines for tolerance regions of ±0.5 and ±1.0 D, respectively. The magenta dashed line indicates the linear regression line, which is very close to the perfect match. The root-mean-squared deviations from the perfect match (RMSE = 0.375 D) and from the linear regression line are listed in the plot (RMSE = 0.379 D) together with the parametric definition of the linear regression line (*y* ~ *a *۰ *x* + *b*) and the coefficient of determination (*R*² = 0.993). D dioptres, RMSE root mean square error.

Figure 3 shows the simulated performance of the lens power calculation based on aphakic refraction for the enVista lens. Figure 3a displays the agreement between the ELP calculated with the Castrop formula and the ELP predicted from the present calculation scheme based on aphakic refraction. Figure 3b displays the agreement between the IOLP calculated with the Castrop formula and the IOLP predicted from the calculation scheme based on aphakic refraction. As can be seen upper right of Fig. 3b, the IOLP calculated from aphakic refraction shows a very mild trend towards underestimation for high-powered lenses.

## Discussion

In cataract surgery, IOLP is generally derived from biometric data using empirical approaches (regression or AI-based), vergence formulae or ray-tracing methods [3, 5, 6]. Unlike empirical strategies [3], vergence and ray-tracing techniques rely on a pseudophakic eye model that may incorporate either a thin- or two-surface cornea and an IOL. Although these calculations use biometric measurements of the phakic eye, the axial position of the implanted lens (ELP) still requires empirical prediction [1, 2, 4].

In rare situations where the AL cannot be measured, conventional IOL calculation methods cannot be applied, and instead, lens power must be estimated from the aphakic refraction [6, 10–12, 19, 20]. Aphakic refraction may be obtained either subjectively or objectively when planning secondary implantation in an aphakic eye or intraoperatively during lens exchange or cataract surgery once the IOL or crystalline lens has been removed [21–23].

Conceptually, IOL planning based on aphakic refraction resembles the calculation for phakic or add-on lenses: the vergence deficit measured at the spectacle plane is transferred to the intended lens plane. In phakic or pseudophakic eyes, the measured ACD provides a basis for estimating the ELP; however, in aphakic eyes—particularly when the AL is unavailable—standard ELP prediction methods cannot be applied.

In the literature, several concepts can be found for lens power calculation based on aphakic refraction, most of which are based on using fixed conversion factors or regressions to translate the vergence deficit at the spectacle plane into a lens power [16, 24–27]. For example, the well-known Ianchulev formula from 2005 [24], based on 22 eyes with intraoperative aphakic refraction, derived a fixed scaling factor of 2.01449 to translate the vergence deficit (mostly equivalent to aphakic refraction at the spectacle plane) into an IOLP. In 2007, Leccisotti [26] showed that for piggyback lenses implanted in the sulcus ciliaris, the IOLP for eyes with an AL > 22 mm could be estimated from the aphakic refraction (with a VD = 13.1 mm) by IOLP = 1.3 ∙ refraction + 1 D. In 2008, Leccisotti used a quadratic regression (*y* = 1.22 + 1.27 ∙ *x* + 0.07 ∙ *x*²) to estimate the IOLP from aphakic refraction measured with autorefractometry [25]. In 2023, Jafarinasab et al. [28] modified this scaling factor to 1.7 and attested a better performance compared with the Ianchulev formula.

However, it is known from vergence calculations that the conversion of aphakic refraction (or vergence deficit) into IOLP depends on the VD, the corneal power and the axial lens position [6, 21, 27, 29]. In a simplified thin lens cornea model, the corneal power could be expressed in terms of a measured corneal front surface radius and a keratometer index. However, in general, corneal power has to be described by the corneal front and back surface radii Ra and Rp, together with the ‘real’ corneal refractive index and the CCT [27].

This simulation study used a modern, fully disclosed vergence formula based on a cornea model with two refracting surfaces as the reference for developing a concept to predict the ELP from the aphakic refraction. To achieve this, the aphakic refraction at the spectacle plane (at a VD in front of the anterior corneal vertex) was back-calculated using a rigorous vergence transform, as described in step A of the present calculation scheme. Similarly, AL can be derived from the aphakic refraction by forward-tracing a paraxial ray bundle through the cornea to its posterior vertex, following the procedure in step B [6, 12, 30].

The back-calculated aphakic refractions were then mapped to the ELP values obtained from the Castrop formula using a large dataset uploaded to IOLCon between 2017 and 2025 for lens constant optimisation (covering 169 different IOL models).

Several ELP prediction models were evaluated, including linear, quadratic, sigmoidal and piecewise linear functions. As shown in Fig. 1, the linear and quadratic models do not capture the saturation behaviour in very short and very long eyes. The one-breakpoint piecewise model performs better, but still overestimates the ELP decrease in short eyes. In contrast, both the sigmoidal model and the two-breakpoint piecewise model reproduce the steep central slope and the flatter behaviour in the extremes. The sigmoidal model was selected because it provides comparable accuracy while offering a simpler, global representation. To account for lens-specific differences, a linear correction term was added based on the *A* constant, derived from the large IOLCon dataset.

Based on the ELP predicted from REFaphakic and the *A* constant, the IOLP may be derived by transforming both the aphakic refraction and the postoperative TR to the predicted ELP plane (difference of both vergences) as outlined in step (D) of the present calculation scheme. In a similar way, the postoperative refraction at the spectacle plane may be predicted by forward transform of REFaphakic to the predicted ELP, considering the power of the implanted lens and backward transform of the resulting vergence to the spectacle plane, as outlined in step (E) of the calculation scheme.

In the absence of real aphakic refraction measurements, the aphakic refraction at the spectacle plane was back-calculated using a simplified aphakic eye model [5] incorporating AL, a two-surface cornea model (Ra, Rp, CCT) and the VD. In aphakic eyes, the refractive error is typically large, which makes VD a critical factor: even small changes in VD can produce substantial shifts in spectacle refraction. As an example, with an aphakic spectacle correction of +12.00 D, the respective refraction transformed to the corneal plane yields a value of +14.02 D for a VD of 12 mm. With a shift of just ±1 mm (VD of 11/13 mm), the corresponding results are +13.82/+14.22 D. Therefore, an accurate VD measurement is essential for any vergence-based calculation in aphakia.

Unlike simple scaling factors or regression-based formulae—which collapse the aphakic refraction into a single empirical multiplier—the present method performs a full vergence calculation using a physically valid cornea model with two refractive surfaces. This allows direct incorporation of the measured anterior and posterior corneal radii and CCT, and eliminates any dependence on a keratometer index. As a result, the approach remains mathematically correct even in eyes with surgically altered anterior corneal curvature, where traditional scaling methods systematically fail. Furthermore, because ELP is predicted solely from the aphakic refraction, the model avoids geometric cross-talk with front-surface changes and maintains robustness after refractive corneal procedures. However, special situations of IOLP calculation such as cataract surgery in eyes with a history of laser vision correction, implantation of the lens in the sulcus or, fixated to the iris, or sutures to the sclera might require a fine-tuning of the lens constant or a strict consideration of a meniscus lens cornea model, taking into account the corneal front and back surface curvature together with CCT [31–33].

As seen from Figs. 2 and 3, even though the mapping of the ELP derived from REFaphakic to the ELP derived with the Castrop formula [1, 2, 4] is not perfect for both lenses, the IOLP prediction derived with the present sigmoidal ELP prediction model and the correction with the *A* constant shows a high degree of match to the IOLP derived with the Castrop formula. This suggests that this concept is a viable subject for clinical evaluation in subsequent studies. Also, independently, the predicted AL as derived with step (B) of the current calculation scheme could be compared with the direct AL measure. However, one should be aware that a direct AL measurement could be affected by a group refractive index of the eye as implemented in most optical biometers, which could make an adjustment (especially in long eyes), e.g., with the ‘sum of segments’ model [34, 35] necessary.

However, this study has some limitations. Firstly, as direct measurements of aphakic refraction (e.g., intraoperative refractometry with a hand-held autorefractor or wavefront sensor) were not available, a back-calculated refraction from an aphakic eye model with two-surface cornea was used as a proxy. In a proof of concept with clinical data, it is recommended that ‘real’ aphakic refraction measurements be used instead. Secondly, the ELP prediction was split into a general model (to be used for all lens types) and an offset correction to consider the specific characteristics of a lens model (using the *A* constant). Even though this concept was derived from a very large and clean dataset, which has been used for lens constant optimisation of many lens models with IOLCon, this concept could be improved with real measurements of the axial lens positions and probably upgraded with individual lens design data. And last but not least, there may be some situations in which the simplistic ELP prediction model based only on aphakic refraction may not be sufficient—for example, in cases where corneal power is affected by an abnormal front-to-back curvature ratio or thickness.

This study proposes a fully vergence-based IOL calculation concept driven solely by aphakic refraction. By using a cornea model with two refracting surfaces and an ELP prediction independent of corneal geometry, the method remains robust even after corneal refractive surgery. Aside from minor limitations in extreme eye lengths, it enables direct prediction of IOLP, postoperative refraction and even AL. Clinical validation will determine its utility and the validity of the ELP prediction concept in cases where the AL cannot be measured.

## Data Availability

The data that support the findings of this study are not publicly available. The minimal dataset is, however, available upon request from the corresponding author.
